# Early adoption of critical care interventions is unjustifiable without concomitant effectiveness study

**DOI:** 10.1186/s13054-020-03382-8

**Published:** 2020-11-18

**Authors:** Hayley B. Gershengorn

**Affiliations:** 1 Division of Pulmonary, Critical Care, and Sleep Medicine, University of Miami Miller School of Medicine, Rosenstiel Medical Science Building, Rm. 7043B, 1600 NW 10th Avenue, Miami, FL 33136 USA; 2 Division of Critical Care Medicine, Albert Einstein College of Medicine, Bronx, NY USA

In 1962, sociologist Everett Rogers created a framework to describe the diffusion of innovation in which he defined five groups by their enthusiasm to embrace new ideas—innovators, early adopters, the early majority, the late majority, and laggards [1]. As applied to medicine, we can consider innovators to be researchers and early adopters to be early implementers in the clinical setting. Those who are slower to embrace novelty may worry that early adoption before a robust evidence base exists might violate our oath to “first, do no harm.” Critical care will always have its early adopters, however, who are excited to try out new therapies which may save lives. Wherever each of us falls on Rogers’s continuum, as a community we must harness the potential of the early adopters’ enthusiasm to inform our future practice. Specifically, we must demand that all early adoption be accompanied by: (1) high-quality observational studies; (2) a commitment to results dissemination, whether they be positive or negative; and (3) a concerted effort to abandon strategies which, after such study, are found to be of low value (Fig. 1).

**Fig. 1:**
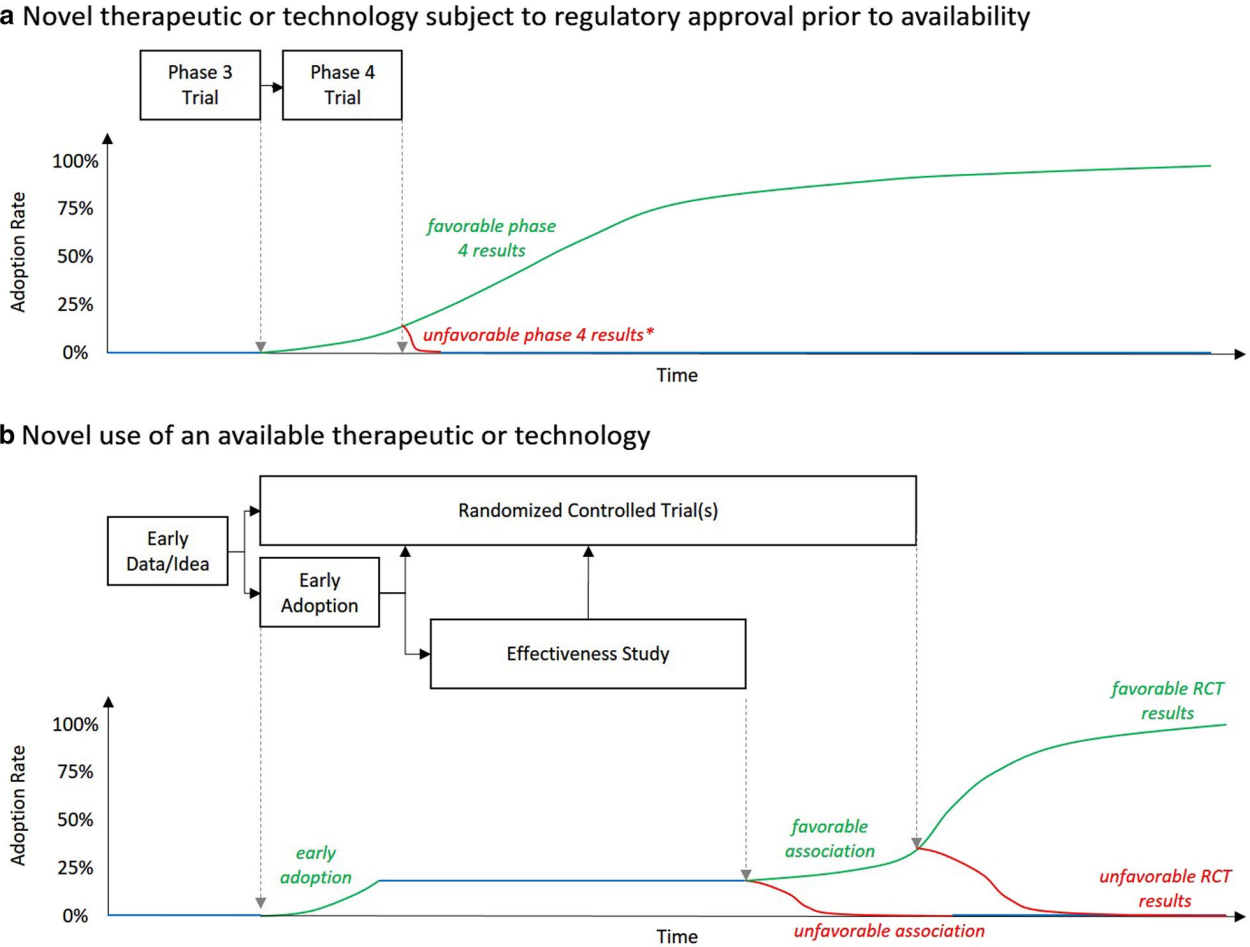
Theoretical timelines for study and adoption/de-adoption of novel interventions. *RCT* randomized controlled trial. Blue line: no change in adoption over time; green line: increasing adoption with time; red line: decreasing adoption with time. *Rapid de-adoption assumed following unfavorable phase 4 trial results under the assumption that regulatory approval would be rescinded rendering the therapeutic/technology unavailable

Within critical care, there are numerous instances of significant early adoption based upon findings from imperfect studies (e.g., tight glucose control [2] or the utilization of hydrocortisone, high-dose ascorbic acid, and thiamine [HAT] for sepsis [3]). This may raise alarm in light of the frequency with which interventions initially thought to hold great promise have been found to be without efficacy (e.g., early goal-directed therapy for sepsis [4] or pulmonary artery catheters [5]) or even harmful (e.g., tight glycemic control [6]) when subjected to higher-quality study. In fact, in the face of COVID-19, many clinicians practiced early adoption of therapies with no evidence behind them; not surprisingly, in some instances, no benefit was found when properly evaluated (e.g., hydroxychloroquine [7]).

Effectiveness studies are of paramount importance to critical care both for therapies adopted early after scant evidence, but also to ensure findings from randomized controlled trials (RCTs) generalize well to the messy, heterogeneity of the real world. When conducted well, effectiveness studies can provide valuable insights which, frequently, are consistent with results from RCTs [8]. As an example, our recent retrospective cohort analysis of > 65,000 adults with septic shock found use of HAT was associated with a 17% increased odds of death, nearly identical in magnitude to the effect found in the VITAMINS RCT [9]. One of the biggest challenges for effectiveness studies is the issue of confounding by indication; however, when use is driven by provider personality rather than patient phenotype, the magnitude of this issue is substantially lessened. While results from observational studies may require confirmation by RCTs, associations suggestive of harm or benefit may alter further adoption and provide needed equipoise to compel trial initiation.

While imperfect, critical care registries can facilitate such study. Absent these, rigorous data collection on patients cared for by both the early adopters and their less-quick-to-act colleagues must be undertaken. With the proliferation of electronic medical records, much needed data may be ascertainable retrospectively; otherwise, prospective collection of, at least, a minimum dataset must be prioritized.

Even with these efforts, however, learning from our early adopters will be challenging as enthusiasm to publish “negative” study results will likely be low. Attempts to mediate publication bias in RCTs (e.g., the development of ClinicalTrials.gov) have not shown great success [10]. Given the absence of similar mandatory registration processes for observational studies, we can expect publication bias to be even greater in this context. Yet, this is an opportunity for the early adopters among us; by pushing yourselves to present the results of effectiveness studies of interventions you have embraced—be they favorable or not—you solidify the importance of your role in our collective betterment. Without early adopters, understanding both the benefits and the harms of novel therapies in the real-world setting before widespread adoption occurs would be retarded.

Finally, if study reveals interventions are without value or, worse, associated with harm, use must cease. This seems straightforward, yet evidence suggests it will not be. As context, the critical care community as a whole is notably poor at achieving widespread use of proven interventions. Take, for example, the case of low-tidal volume ventilation for acute respiratory distress syndrome, a therapy with mortality benefits proven by a high-quality RCT [11]. Nearly a decade after publication, compliance at multiple sites remained poor, with fewer than 3 in 10 patients receiving the intervention [12, 13]. De-adoption is even more challenged. In the face of new evidence suggesting non-value or even harm, critical care clinicians are slow and incompletely willing to abandon current paradigms of care [2, 14]. Early adopters—some found to be “impulsive”, but others, “reflexive” (cautiously evaluative of new technologies) [15]—may be differently primed to de-adopt than the critical care community at large. Together, we must push ourselves to aggressively implement what we know is useful and to actively give up that which we have learned is not.

Critical care clinicians are, in some ways, as diverse as the patients for whom we provide care. Our heterogeneity in enthusiasm for potential new therapies is often apparent even within individual intensive care units. This is reality. Sometimes it may cause tension, yet it also provides opportunity. To harness this potential, however, requires a commitment to high-quality effectiveness research, near-complete results dissemination, and concerted efforts to improve both implementation of effective therapies and de-adoption of all others. Without this, early adoption—especially without high-quality preliminary trial data—is unjustifiable.

## Data Availability

Not applicable.
